# Adenocarcinoma of the Ampulla of Vater: A Case Report on a Rare Condition

**DOI:** 10.7759/cureus.29398

**Published:** 2022-09-21

**Authors:** Vaishnavi V Kantode, Ranjana Sharma, Mayur B Wanjari, Pratiksha K Munjewar, Suhas P Tivaskar, Abhas Nakhale

**Affiliations:** 1 Medical Surgical Nursing, Smt. Radhikabai Meghe Memorial College of Nursing, Datta Meghe Institute of Medical Sciences, Wardha, IND; 2 Research Scientist, Jawaharlal Nehru Medical College, Datta Meghe Institute of Medical Sciences, Wardha, IND; 3 Radiology, Datta Meghe Institute of Medical Sciences, Wardha, IND; 4 Community Health Nursing, Smt. Radhikabai Meghe Memorial College of Nursing, Datta Meghe Institute of Medical Sciences, Wardha, IND

**Keywords:** whipple procedure, cholangiopancreatography, carcinoma, duodenum, s: ampulla of the vater

## Abstract

An ampullary carcinoma (AC) is defined as cancer that arises in the ampulla of Vater (AV). It is a tiny opening in the beginning segment of the small intestine called the duodenum. Pancreatic and bile duct fluids are pumped into the intestines via the AV. There are various factors listed as the causes of AC. A 45-year-old male presented to the emergency department with complaints of pain in the abdomen for two months, vomiting for two days, and a history of fever for four days, which was persistent in nature. He had been operated on for endoscopic retrograde cholangiopancreatography (ERCP) stenting two months back due to pain in the abdomen. The patient underwent investigations such as blood tests, histopathology, ultrasonography, abdominal contrast-enhanced computed tomography (CECT), and ampullary mass biopsy, based on which a final diagnosis was made. The patient was operated on by the Whipple surgical procedure and was later treated with antibiotics and analgesics. Surgical management for AC is a novel treatment chosen instead of chemotherapy. Although early-stage AC can be cured with radical surgery, around half of the patients experience tumor recurrence. The prognosis of our patient was good.

## Introduction

Ampullary carcinomas (AC) are rare entities, accounting for only 0.2% of gastrointestinal cancers and approximately 7% of all periampullary cancers. They arise from the ampullary complex, distal to the confluence of the common bile and pancreatic duct [1]. Adenocarcinomas account for the majority of ampullary carcinomas. However, the histology differs among the subtypes such as papillary, adenosquamous, mucinous, and adenocarcinomas [2]. Further research is required to evaluate the role of contemporary, multi-agent chemotherapy and chemoradiotherapy in patients with resected and advanced ampullary adenocarcinoma. Adjuvant chemotherapy is favored for adenocarcinoma over observation alone.

## Case presentation

The patient was a 45-year-old male who presented to the emergency department with complaints of pain in the abdomen for two months, which had been insidious in onset and gradually progressive in nature and non-bilious, non-projectile vomiting for two days with five episodes per day, and a history of fever with an insidious onset that had persisted for the last four days. He was a chronic alcoholic and smoker for 20 years.

**Figure 1:**
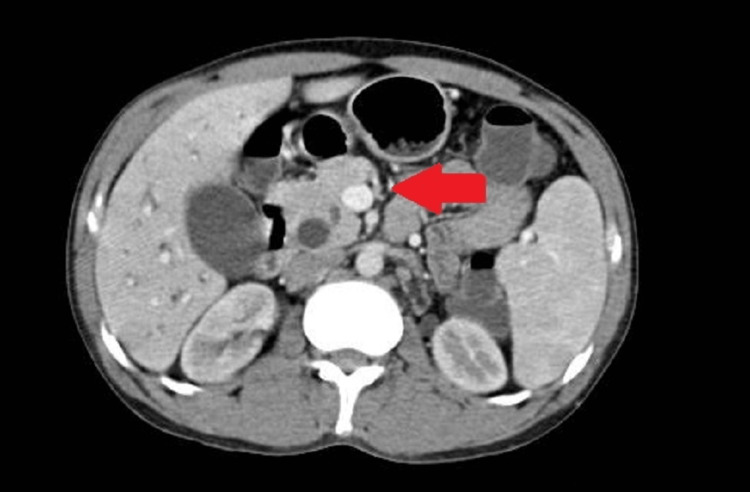
An ill-defined hypodense minimally enhancing lesion of 1.8 x 1.2 cm in the ampullary region (arrow)

The patient had a history of pain in the abdomen and reduced appetite two months back. Contrast-enhanced computed tomography (CECT) of the abdomen was done, which revealed dilated common bile duct (CBD) of 12 mm, hepatic duct, and intra and extrahepatic biliary radical with dilated main pancreatic duct (MPD) of 4.5 mm. There was evidence of an ill-defined hypodense minimally enhancing lesion of 1.8 x 1.2 cm in the ampullary region causing abrupt narrowing of distal CBD and MPD (Figure 1). For the indication of cholangitis due to an ampullary mass, he underwent endoscopic retrograde cholangiopancreatography (ERCP). It showed obstructive jaundice and, ampullary mass precut accessotomy was done. A 10-cm straight plastic stent was inserted into CBD to drain the free flow of bile, and a biopsy was obtained and sent to histopathology for analysis. Histopathology report raised suspicion of well-differentiated adenocarcinoma of the ampulla. A diagnosis of adenocarcinoma of the ampulla of Vater (AAV) was made. After this procedure, the patient was discharged in three days and he planned to undergo a surgical procedure for the removal of the tumor. He was again readmitted for the above-mentioned problems after two months.

On arrival, a physical examination was done, and tenderness was noted in the abdomen's epigastric and right lumbar region and tympanic note on percussion. On radiological investigations, the ultrasonography of the abdomen and pelvis showed hepatomegaly of 16.5 cm in size with altered echotexture and splenomegaly of 16.6 cm in length. After this investigation, the patient was advised to undergo a CECT of the abdomen to rule out periampullary region pathology. Abdominal CECT revealed that the head of the pancreas had an isodense-enhancing mass lesion of around 4.3 x 3.7 cm in size. These findings pointed to periampullary cancer with obstructive dilatation of CBD, common hepatic duct (CHD), intrahepatic biliary radical (IHBR), and liver lobes.

A laboratory examination was done on admission, which revealed more elevated biliary enzymes (Table 1).

**Table 1:** Laboratory investigations on liver function

Investigation	Normal value	Patient value
Alkaline phosphate	44–147 IU/L	1364 IU/L
Serum glutamic-pyruvic transaminase (SGPT)	7–56 U/L	172 U/L
Serum glutamic-oxaloacetic transaminase (SGOT)	8–45 U/L	154 U/L
Total bilirubin	1.2 mg/dl	7.8 mg/dl
Lipase	0–160 U/L	333 U/L

The physician planned for a Whipple surgery (pancreaticoduodenectomy). After the surgery, the patient was shifted to the surgical ICU and kept nil by mouth till further orders; he was placed in a propped-up position of up to 30 degrees, and intravenous fluids were advised to be administered at a rate of 100 ml/hour. Also, he was put on antibiotics (injection of ceftriaxone 1.5 gm, metronidazole 100 ml, and amikacin 500 mg) and injection of Neomol 100 ml. The prognosis of the patient was good.

## Discussion

AC is a malignancy that develops in the ampulla of Vater (AV). AV is a tiny aperture in the small intestine's initial segment, also known as the duodenum. It transports pancreatic and bile duct fluids into the intestines [3]. Ampullary cancers are not very common and account for 0.2% of gastrointestinal malignancies and 7% of all periampullary cancers. They emerge from the ampullary complex, located near the junction of the common bile and pancreatic ducts [4]. Since 1973, the incidence of ampullary carcinoma has been on the rise. In both African Americans and Caucasians, the disease is more common in men [5].

The pathogenesis of AC is unknown, but there may be a link with a non-invasive component that follows the same adenoma-to-carcinoma sequence as colorectal carcinoma [6]. It becomes more common as people get older, and individuals who are treated surgically have considerably better outcomes. Ampullary cancer can also be discovered and treated significantly sooner using endoscopic procedures [7]. Carcinoma of the AV is more resectable and has a greater prognosis than pancreatic cancer [8].

AC can have various signs and symptoms. Jaundice is the most typical sign, exhibited by 82% of the patients. It also manifests as yellowish skin and eyes, clay-colored stools, pain in the abdomen, malaise, rectal bleeding, nausea, vomiting, and weight loss [9]. Various diagnostic methods can be used to confirm the existence of an ampullary tumor. Endoscopy, endoscopic ultrasound, abdominal ultrasonography, CT, MRI, and positron emission tomography are used to aid in detecting and staging these tumors [10].

The most common surgery is a pancreaticoduodenectomy, a complicated operation also called the Whipple procedure, where the tumor is removed from the affected part of the AV and the surrounding area [11]. The patient's postoperative survival increases by 10-20% following the Whipple resection [12].

The Whipple procedure can lead to some specific complications. Following are some of the most severe postoperative problems associated with this procedure: a fistula or a leak in the pancreas, abscess within the abdomen, leakage of bile, hemorrhage occurring postoperatively, which requires blood transfusion or reopening, emptying of the stomach being delayed, infection to the surgical site, and wound dehiscence [11-12].

## Conclusions

AAV is a very unusual form of carcinoma; it is a life-threatening diagnosis but can be treated with surgery called the Whipple procedure. Another name for the Whipple procedure is pancreaticoduodenectomy. AC has a better outcome and excellent prognosis than pancreatic cancer. Timely detection and diagnosis may help to influence survival. The cause of AC is unknown, but it is hypothesized that abnormal cell growth creates lumps, masses, and tumors. It can be managed surgically, and the survival rate has also improved as per studies in the literature.
